# LIPUS-S/B@NPs regulates the release of SDF-1 and BMP-2 to promote stem cell recruitment-osteogenesis for periodontal bone regeneration

**DOI:** 10.3389/fbioe.2023.1226426

**Published:** 2023-07-04

**Authors:** Shujin Yan, Dong Wang, Liang Zhang, Tian Gan, Huan Yao, Hui Zhu, Yiman He, Ke Yang

**Affiliations:** ^1^ Ministry of Education Key Laboratory of Child Development and Disorders, Pediatric Research Institute, National Clinical Research Center for Child Health and Disorders, China International Science and Technology Cooperation Base of Child Development and Critical Disorders, Chongqing Engineering Research Center of Stem Cell Therapy, Children’s Hospital of Chongqing Medical University, Chongqing, China; ^2^ Department of Ultrasound, The First Affiliated Hospital of Chongqing Medical University, Chongqing, China; ^3^ Department of Ultrasound, Women and Children’s Hospital of Chongqing Medical University, Chongqing, China

**Keywords:** LIPUS, nanoparticle, stem cell, recruitment-osteogenesis, periodontal bone regeneration

## Abstract

**Purpose:** Poly (lactic-co-glycolic acid)-based nanoparticles (PLGA NPs) have been widely used as the carrier for sustainable drug delivery. However, the drug release from the NPs was usually incomplete and uncontrollable. Herein, a low intensity pulsed ultrasound (LIPUS) assisted SDF-1/BMP-2@nanoparticles (S/B@NPs) system was fabricated to facilitate stem cell recruitment-osteogenesis for periodontal bone regeneration.

**Methods:** In this work, S/B@NPs were prepared with double-emulsion synthesis method. Then the S/B release profile from NPs was evaluated with or without low intensity pulsed ultrasound treatment. Afterwards, the stem cell recruiting and osteoinductive capacities of LIPUS-S/B@NPs were detected with human periodontal ligament cells (hPDLCs) *in vitro* and in a rat periodontal bone defect model.

**Results:** The results indicated that S/B@NPs were successfully prepared and LIPUS could effectively regulate the release of S/B and increase their final releasing amount. Moreover, LIPUS-S/B@NPs system significantly promoted hPDLCs migrating and osteogenesis *in vitro* and recruiting rBMSCs to the rat periodontal defect and facilitated bone regeneration *in vivo*.

**Conclusion:** Our LIPUS assisted S/B@NPs system can effectively facilitate stem cell recruitment and periodontal bone regeneration. Considering its reliable safety and therapeutic effect on bone fracture, LIPUS, as an adjuvant therapy, holds great potential in the regulation of drug delivery systems for bone healing.

## 1 Introduction

Periodontitis is a common chronic disorder affecting health and life quality of adults (Kinane et al., 2017). Ultimately, it leads to irreversible destruction of alveolar bone due to the continuous chronic inflammation (Huang et al., 2021). In some advanced cases, it even leads to tooth loss. Nowadays many studies have focused on the regeneration of periodontal tissues. However, the effective treatments to repair the destructed periodontal bone tissues are still limited (Silva et al., 2019).

These years, stem cell therapy has emerged as one of the mainstream strategies for periodontal regeneration (Chen et al., 2012). Among various kinds of stems cells, human periodontal ligament cells (hPDLCs) with high osteogenic potential have been considered an ideal stem cell for bone tissue engineering (Zhao et al., 2022). Some researchers implanted hPDLCs loading scaffold to the damaged periodontal bone defect and achieved a certain degree of periodontal bone regeneration (Han et al., 2014). However, the tedious work of obtaining and culturing stem cells and the low survival rate of exogenous cells delivered, as well as the potential risk of infections from the donor greatly limit its application (Chen and Jin, 2010).

*In situ* tissue regeneration proposes a new conception of recruiting and utilizing the resident stem cells of the host for tissue regeneration (Safina and Embree, 2022). To this end, stromal cell-derived factor-1 (SDF-1) from the CXC chemokine family plays a key role in recruiting CXCR4 (CXC motif chemokine receptor type 4)-positive stem and progenitor cells in the early stage of tissue repairment (Yu et al., 2020; Ling et al., 2022). Therefore, the exogenous delivery of SDF-1 holds great potential in the process of *in situ* bone regeneration. After the cell recruitment, the next most important thing is to initiate their osteogenesis for further bone regeneration (Wang et al., 2023). BMP is a kind of growth factor derived from demineralized bone matrix and plays a key role in inducing bone formation (Lowery and Rosen, 2018). BMP-2 is one of the most important osteogenic growth factors in the BMP family. It has been applied for clinical fracture therapy and its ability to promote osteogenic differentiation of MSC has been demonstrated in many studies (Lo et al., 2012; Hasenbein et al., 2022).

Therefore, BMP-2 may provide an effective bio-signal for osteogenesis of the recruited stem cells. In clinic, growth factors are usually directly injected into the defect area to promote bone healing, which may cause their rapid loss in the targeted area (Wink et al., 2014; de Freitas et al., 2016). Moreover, bioactive molecules are generally unstable and easily degraded *in vivo* (Dumic-Cule et al., 2018), and high concentrations of BMP-2 may lead to many side effects such as ectopic osteogenesis, local inflammation and hemangioma (Wang B. et al., 2018a). Therefore, biomaterial-based carriers are needed for the incorporation and delivery of biomolecules to maintain the bioactivity of the factors and sustain their release at the targeted area (Wang et al., 2022). Poly (lactic-co-glycolic acid)-based nanoparticles (PLGA NPs) have been widely used as the carrier for drug delivery with the good biocompatibility and biodegradability of PLGA (Paul et al., 2022; Rocha et al., 2022). PLGA NPs based drug release was controllable compared to the traditional direct drug injection. However, such controllable drug release is limited because it simply delayed the release of drugs (Sawant et al., 2021).

Ultrasound, as a physical stimulus, has been widely utilized to initiate and regulate the release of drugs from their carriers in the targeted area (Li et al., 2021). However, ultrasound with excessive intensity may cause damage to bone healing, like thermal effect. As a physical therapy for bone fracture, low intensity pulsed ultrasound (LIPUS) has been widely adopted for bone healing in clinic (Yao et al., 2022). Moreover, it has been reported to promote cell migration and osteogenic differentiation. However, whether it is able to regulate the drug release from the PLGA NP carriers is unknown (Liu et al., 2020).

**FIGURE 1:**
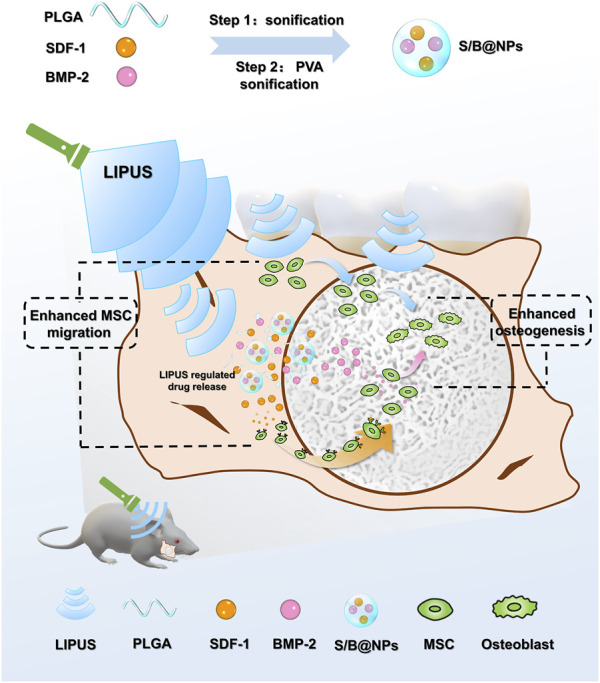
Schematic diagram depicting the design principle of the biomimetic LIPUS assisted S/B@NPs-delivery system recruiting and facilitating the osteogenesis of stem cell in rat periodontal defect area under the radiation of LIPUS.

Herein, a PLGA NP-based drug delivery system was fabricated, which conducted controlled releases of SDF-1 and BMP-2 under LIPUS radiation. In this system, the dual release of SDF-1 and BMP-2 biomolecules was set to couple the stem cell recruitment and osteogenesis processes, and the combination of LIPUS was able to optimize the release of the biomolecules in this system while promoting bone healing with its physical therapeutic effect. The cell recruitment and osteoinductive capacities of this system were evaluated *in vitro* and in a rat periodontal defect model. Our research is expected to lay a theoretical foundation for the development of LIPUS assisted drug delivery system in bone tissue engineering in the future (Figure 1).

## 2 Materials and methods

### 2.1 Synthesis of S/B@NPs

SDF-1/BMP-2@PLGA nanoparticles (termed S/B@NPs) were prepared by double-emulsion synthesis method (Liu et al., 2021). 50 mg PLGA (Ruixi Biological Technology, Xi’an, China) was added into 2 mL dichloromethane and vortexed to make it totally dissolved. SDF-1 and BMP-2 (Novaprotein) were dissolved in 1% bovine serum albumin (BSA, Sigma) solution and then it was added into the PLGA solution. To obtain the primary emulsion, the protein-containing solution was emulsified with sonicator (Sonics and Materials Inc., Newtown, Connecticut, United States) under ice bath for 2 min. Then 9 mL of 4% PVA solution was supplemented to it and sonicated for another 2 min. W/O/W double emulsion was so far formed. 10 mL of 2% isopropanol solution was added into the emulsion. After further magnetic stirring for 4 h and centrifugation for 5 min (10,000 rpm), the NPs were harvested and reserved for further evaluation.

### 2.2 Characterization of S/B@NPs

The transmission electron microscope (TEM, Hitachi, Tokyo, Japan) and scanning electron microscopy (SEM, Hitachi, Tokyo, Japan) were employed to measure the morphology and structure of the S/B@NPs. Afterwards, a Zetasizer Nano ZS instrument (Zetasizer Nano ZS90, Malvern Instruments, United Kingdom) was used to detect their hydrodynamic diameters and zeta potentials.

### 2.3 The protein entrapment efficiency of NPs

Bovine serum albumin (BSA, Solarbio, Beijing, China) was used as the model protein of SDF-1 and BMP-2 because of its good stability and low cost. The protein entrapment efficiency was evaluated by Micro-BCA method with the BCA kit (Beyotime, Shanghai, China). The 0.9 mL of NaOH (1 mol/L) and 0.1 mL phosphate-buffered saline (PBS) were mixed, then 5 mg of BSA@NPs was added to the mixture and shaken at room temperature. After 2 h of hydrolysis, the aforementioned solutions were neutralize using 1 mL of HCl (0.9 mol/L). Following Micro-BCA assay was performed to assess the protein concentrations using the BCA kit.

### 2.4 Release of SDF-1/BMP-2 from NPs under LIPUS stimulus

S/B@NPs were dissolved in PBS. Then the samples were equally divided as control and LIPUS-treated groups. The samples in LIPUS group were exposed to LIPUS irradiation (JC 200, Chongqing Haifu Technology, Chongqing, China) at a frequency of 3 MHz, an intensity of 100 mW/cm^2^, a duty cycle of 50% for 10 min every 6 h. The tubes were placed under 37°C shaker bath (Memmert WB14, Schwabach, Germany) at 100 rpm for 120 h. Their supernatants were collected from each tube at each timepoint. The contents of SDF-1 and BMP-2 were evaluated according to the manufacturer’s instructions with the ELISA kit (Jiubang Biotechnology Quanzhou, China). Then the release profiles were finally obtained by plotting the percentages of cumulative contents of the released SDF-1 and BMP-2 as time.

### 2.5 *In vitro* cytotoxicity assay

The cytotoxicity of NPs was measured by Cell Counting Kit-8 (CCK-8) assay. Cells were seeded in a 96-well plate and incubated with NPs of different concentrations (50, 125, 500, 1,000, and 2,000 μg/mL) for 1 and 3 days. At each timepoint, 10 µL of CCK-8 (Dojindo, Kumamoto, Japan) solution was added into each well. After 2 h incubation, the plate was placed in a microplate reader (Synergy HT, BioTek, Winooski, VT) to measure their absorbance at 450 nm. The biocompatibility of the NPs was further assessed with live/dead assay for 1 and 3 days. Briefly, cells were seeded in glass cell culture dishes and incubated with or without aforementioned concentrations of NPs. On the first and third day of incubation, the cells were stained using calcein-AM/PI kit (Beyotime), after which the cells were observed under a laser scanning confocal microscope (Nikon, Tokyo, Japan).

### 2.6 Cell isolation and culture

The hPDLCs were donated by Professor Jinlin Song from the Stomatological Hospital of Chongqing Medical University. 10% fetal bovine serum (FBS; Gibco, United States), 100 U/mL of penicillin and 100 μg/mL of streptomycin were supplemented to the α-Minimum Essential Medium (α-MEM; Hyclone, Logan, UT, United States) to form the complete medium for cells culture. The cells were cultured at 37°C in a humidified CO_2_ incubator. When reaching 60% confluency, the cells were passaged. The following experiments were conducted with cells of passage 3.

To investigate the osteoinductivity of our system, four groups were set: 1) control group, the cells were cultured in osteogenic differentiation medium (complete medium further supplemented with 50 μg/mL ascorbic acid, 10 mM *ß*-glycerophosphate, 10 nM dexamethasone (Solarbio, Beijing, China)); 2) the S/B group, the cells were incubated in osteogenic differentiation medium with SDF-1 and BMP-2; 3) the S/B@NPs group, the cells were incubated in osteogenic differentiation medium containing S/B@NPs; 4) the LIPUS + S/B@NPs group, the cells were cultured in osteogenic differentiation medium with S/B@NPs under LIPUS radiation at a frequency of 3 MHz, an intensity of 100 mW/cm^2^, a duty cycle of 50%, for 10 min every day.

### 2.7 Alkaline phosphatase (ALP) and alizarin red staining

ALP and alizarin red staining were conducted to assess the cell osteoinductive capacity of our system. After 7 days of osteogenic induction, the hPDLCs were fixed and stained according to the instructions of ALP staining kit (Beyotime). After staining, images were obtained under the microscope (Nikon Eclipse Ti, Tokyo, Japan). For the purpose of further evaluating its osteoinductive capacity, alizarin red solution (Solarbio) was used to detect the calcium accumulation in cells after 21 days of the osteogenic induction. After incubation for 30 min, the samples were imaged under the microscope. Then the 10% cetylpyridinium chloride (Macklin, Shanghai, China) was used to quantify calcium accumulation. The solution was then shifted to a 96-well plate, and then their optical density was detected at 405 nm under the microplate reader.

### 2.8 Transwell migration assay

The influence of our system on hPDLCs migration was evaluated in a 24-well transwell system (pore size: 8 μm; Merck, United States). Firstly, hPDLCs were starved for 3 h and harvested by trypsinization. Then the cells were seeded in the upper chambers of the transwell system (1 × 10^5^ cells per chamber). The lower chambers were treated with serum-free media (Control group) or different groups (S/B group, S/B@NPs group, LIPUS + S/B@NPs group). After 24 h, 0.2% crystal violet (Solarbio) was used to stain the hPDLCs migrated to the bottom side of transwell and the microscope was employed to take the images of stained hPDLCs.

### 2.9 Analysis of mRNA expression

After 7-day osteoinductive culture, RNAsimple total RNA kit (Tiangen Biological Technology, Beijing, China) was used to extract the cells’ total RNA. Afterwards, Reverse transcription kit (Thermo Fisher Scientific, America) was applied to synthetize cDNA. Real-time PCR was conducted to evaluate the alkaline phosphatase (*ALP*), runt-related transcription factor 2 (*Runx2*), and collagen type I (*Col1a1*) mRNA expressions of hPDLCs in Real-Time PCR Detection (Bio-rad). *GAPDH* expression was used as the internal control. The primer sequences of aforementioned genes were listed in Table 1.

**TABLE 1:** The primer sequences of all genes used in qPCR.

Gene	Forward primer (5′-3′)	Reverse primer (5′-3′)
*ALP*	ACC​ACC​ACG​AGA​GTG​AAC​CA	CGT​TGT​CTG​AGT​ACC​AGT​CCC
*Runx2*	TGG​TTA​CTG​TCA​TGG​CGG​GTA	TCT​CAG​ATC​GTT​GAA​CCT​TGC​TA
*Col1a1*	GAG​GGC​CAA​GAC​GAA​GAC​ATC	CAG​ATC​ACG​TCA​TCG​CAC​AAC

### 2.10 Immunofluorescence staining

The protocol of immunofluorescence staining was consistent with that reported in previous studies (Yao et al., 2022). Briefly, the hPDLCs were seeded in glass cell culture dishes used for laser scanning confocal microscopy. After treating for 5 days, the 4% PFA was used to fix the hPDLCs, and then they were permeabilized with 0.5% (v/v) Triton X-100 (Solarbio). After that, the hPDLCs were blocked employing 5% BSA. Then the anti-Runx2 (rabbit monoclonal antibody, 1:50, Abcam) solution was used to incubate the cells at 4°C overnight. Afterwards, the cells were incubated with corresponding secondary antibody (CoraLite488-conjugated goat anti-rabbit IgG, 1:100, Proteintech, Rosemont, IL) at room temperature for 1.5 h, followed by counterstained using 2-(4-amidinophenyl)-6-indolecarbamidine dihydrochloride (DAPI) (1:150, Leagene) for 5 min. Then the laser scanning confocal microscope was applied to observe the cells and images were captured. The images were processed with the ImageJ software (National Institutes of Health, Bethesda, MD, United States).

### 2.11 The preparation of periodontal bone defect model *in vivo*

The animal experiments process was reviewed and approved by the Ethics Committee of Children’s Hospital of Chongqing Medical University. Male Sprague Dawley (SD) rats of 8 weeks old were obtained from Laboratory Animal Center of Chongqing Medical University and randomly divided into 5 groups including control group, Hydroxyapatite/Beta-tricalcium phosphate (HA/β-TCP) group (termed HT group), S/B-HT group, S/B@NPs-HT group and LIPUS + S/B@NPs-HT group. 500 μL S/B@NPs suspension or S/B solution were mixed with the same amount of HT powder in EP tubes, respectively. The mixtures in the EP tubes were gently shaken at 4°C overnight.

According to the protocol reported in the previous study, the periodontal bone defect was prepared after modifications (Padial-Molina et al., 2015; Wang et al., 2023). The rats were anesthetized, and their fur around the operation region was removed, followed by the disinfection of cheek skin. To expose the masseter muscle, an incision with 1.5 cm was made alongside the lower margin of the alveolar bone. After the masseter muscle was dissected and the buccal plate was localized, accession to the buccal roots of molars was initiated with a ring drill and the bone in the defect area was removed with a ball drill. Finally, a 3 mm diameter circular defect was made and implanted with or without drug delivery systems, after which the muscle and skin over the operation area were relocated and sutured. The rats were injected with analgesia once a day for 3 days after the operation, and fed with soft food for 1 week. The LIPUS treatment at a frequency of 3 MHz, an intensity of 100 mW/cm^2^, a duty cycle of 50% was performed 20 min every 2 days to the rats in the LIPUS + S/B@NPs group. After 8 weeks, the rats were sacrificed and their mandibles were collected, fixed and scanned using microcomputed tomography (micro-CT, Always Imaging, Shanghai, China). Then 10% EDTA buffer was adopted for the decalcification of samples for 2 months, and the samples were further sectioned for H&E and Masson trichrome staining.

### 2.12 *In vivo* toxicity of the drug-delivery system

To assess the toxicity of our drug-delivery systems *in vivo*, different groups of systems (NPs-HT group, S/B@NPs-HT group, LIPUS + S/B@NPs-HT group and control group) were implanted to the periodontal defects of SD rats. LIPUS treatment was conducted to the LIPUS + S/B@NPs-HT group. 8 weeks after surgery, the viscus tissues of rats in all groups were collected, fixed and sectioned for H&E staining.

### 2.13 rBMSCs recruitment *in vivo*

To assess the cell recruitment ability of our systems *in vivo*, CFSE staining kit (Invitrogen, California, United States) was employed to mark rat bone marrow mesenchymal stem cells (rBMSCs). The rBMSCs were collected and resuspended in PBS containing the CFSE probe, and the solution were incubated at 37°C. After 15 min, the cells were centrifuged, resuspended in medium and then incubated for another 30 min. Then the cells were observed under the fluorescence microscope and images were captured. The periodontal bone defect model was prepared in rats as described above, followed by the injection of CFSE-labelled rBMSCs into rats’ circulation through the caudal vein. After 1 week, their mandibles were collected, fixed and sectioned with a hard tissue slicing technique. The confocal fluorescence microscope was employed to observe the CFSE-labelled rBMSCs migrated to the periodontal defect area.

### 2.14 Statistical analysis

The data are presented as the mean ± standard deviation (SD) and the differences between groups were evaluated with one-way analysis of variance (ANOVA). The significance level was set to *p* < 0.05, *p* < 0.01, *p* < 0.001 and *p* < 0.0001 for our experimental data analysis. A *p*-value of less than 0.05 was considered statistically significant. The GraphPad Prism 9.0 (GraphPad Software Inc., San Diego, CA, United States) software was used for statistical analysis of the data.

## 3 Results

### 3.1 Preparation and characterization of the S/B@NPs

**FIGURE 2:**
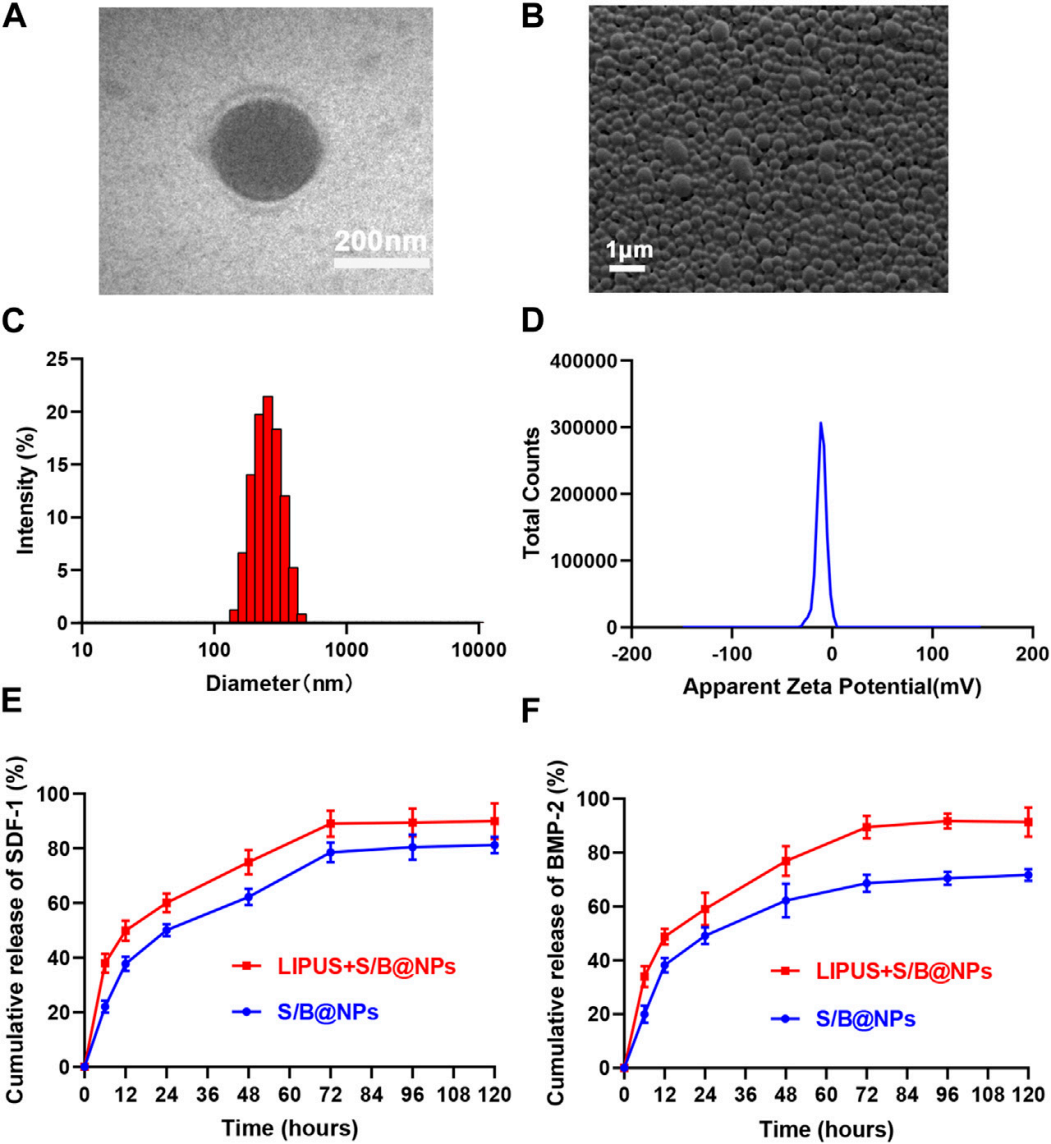
Synthesis and characterization of S/B@NPs. **(A)** TEM image of S/B@NPs. **(B)** SEM image of S/B@NPs. **(C)** Particle size distributions of S/B@NPs. **(D)** Zeta potentials of S/B@NPs. **(E)** Release profiles of the SDF-1 from S/B@NPs with or without LIPUS treatment. **(F)** Release profiles of the BMP-2 from S/B@NPs with or without LIPUS treatment. Error bar represents the mean ± SD (*n* = 3).

As shown in Figure 2A, the core-shell structure and spherical shape of S/B@NPs could be observed under TEM. Under the SEM, it could be seen that the NPs had good dispersion and quite uniform size (Figure 2B). The average diameter and Zeta potential of NPs were 244.6 ± 64.66 nm (Figure 2C) and −9.82 ± 5.15 mV, respectively (Figure 2D). The protein entrapment efficiency of NPs is 51%.

As shown in Figures 2E, F, the LIPUS treated group displayed a higher release rate of SDF-1 and BMP-2 than the control group since the 0th h. Both groups gradually reached a plateau at about 72nd h, by which time the LIPUS group showed a significant higher amount of SDF-1 and BMP-2 released, which indicated that LIPUS could effectively regulate the release of bioactive factors from S/B@NPs.

### 3.2 Biocompatibility evaluation of S/B@NPs *in vitro*

**FIGURE 3:**
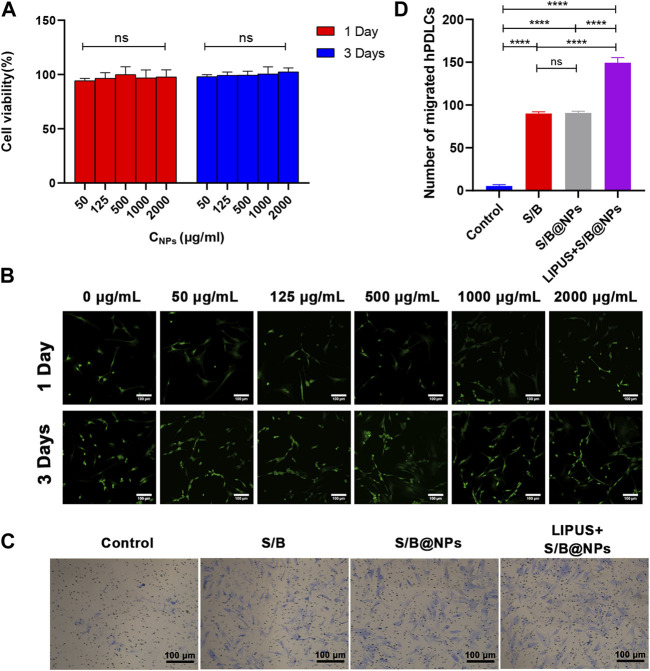
Biocompatibility evaluation of LIPUS-S/B@NPs and its effect on cell migration. **(A)** CCK-8 results of S/B@NPs in day 1 and day 3 of culture. **(B)** Results of Live (green)/Dead (red) fluorescence assay of hPDLCs after 1 and 3 days of co-culture. Scale bar: 100 μm. **(C)** Optical images of hPDLCs recruited in the transwell assay and **(D)** their quantification. Error bar represents the mean ± SD (*n* = 3); *, *p* < 0.05; **, *p* < 0.01; ***, *p* < 0.001; ****, *p* < 0.0001.

To examine the biocompatibility of S/B@NPs, the hPDLCs were cocultured with NPs and CCK-8 assay was employed to detect their cytotoxicity. The cell viability exhibited no significant differences in groups with different concentrations of S/B@NPs on both day 1 and day 3 (Figure 3A). And from 1 day to 3 days, the cells in all groups displayed a good growth trend, which suggested that NPs displayed low cytotoxicity in the concentration from 50 to 2,000 μg/mL. As for the live/dead cell staining, all the experimental groups exhibited a lot of living cells with green fluorescence and rare dead cells with red fluorescence (Figure 3B), and no significant differences were detected among the experimental groups. From day 1 to day 3, the number of living cells significantly increased in all groups. These results were consistent with the CCK-8 results, indicating that 50–2,000 μg/mL NPs had good biocompatibility.

### 3.3 LIPUS-S/B@NPs promoted hPDLCs migratio*n in vitro*

The recruitment of stem cells to the targeted area is the crucial first step for *in situ* bone regeneration. In this study, transwell migration assay was performed to evaluate the influence of LIPUS-S/B@NPs system on the migration ability of hPDLCs. As shown in Figures 3C, D, the numbers of migrated hPDLCs in the S/B group, S/B@NPs group and LIPUS + S/B@NPs group were significant increased than that of the control group. The S/B@NPs group exhibited similar migration activity compared with the S/B group, whereas LIPUS + S/B@NPs group displayed significantly increased number of migrated cells than other groups. These results indicated that our LIPUS assisted S/B@NPs system could effectively promote the migration ability of hPDLCs.

### 3.4 LIPUS-S/B@NPs promoted hPDLCs osteogenic differentiation *in vitro*

**FIGURE 4:**
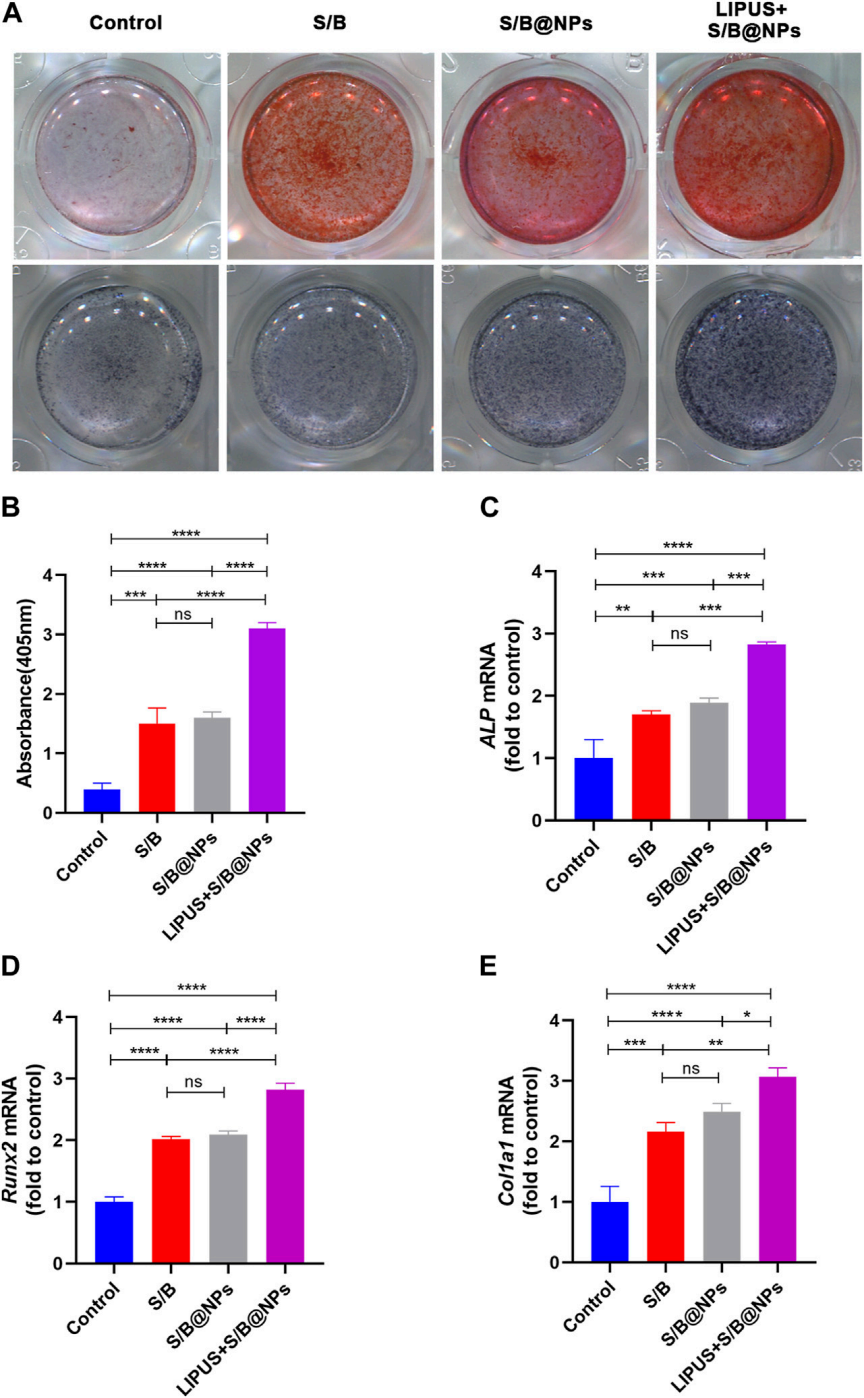
The effect of LIPUS-S/B@NPs on cell osteogenesis. **(A)** ALP staining and alizarin red staining images of hPDLCs. **(B)** Quantification of ARS staining, and gene expressions of **(C)** *ALP*, **(D)** *Runx2*, **(E)** *Col1a1*. Error bar represents the mean ± SD (*n* = 3); *, *p* < 0.05; **, *p* < 0.01; ***, *p* < 0.001; ****, *p* < 0.0001.

To evaluate the osteoinductive capacity of our system, ALP and alizarin red stainings were conducted. After 7 days treatment, the results of ALP staining presented that the cells in all the experimental groups exhibited more positive staining than the control group (Figure 4A). Moreover, the LIPUS + S/B@NPs group showed the deepest staining among all the groups, while no obvious difference was detected among the S/B@NPs and the S/B groups. After culture for 21 days, the results of alizarin red staining were in accordance with those of ALP staining (Figures 4A, B).

**FIGURE 5:**
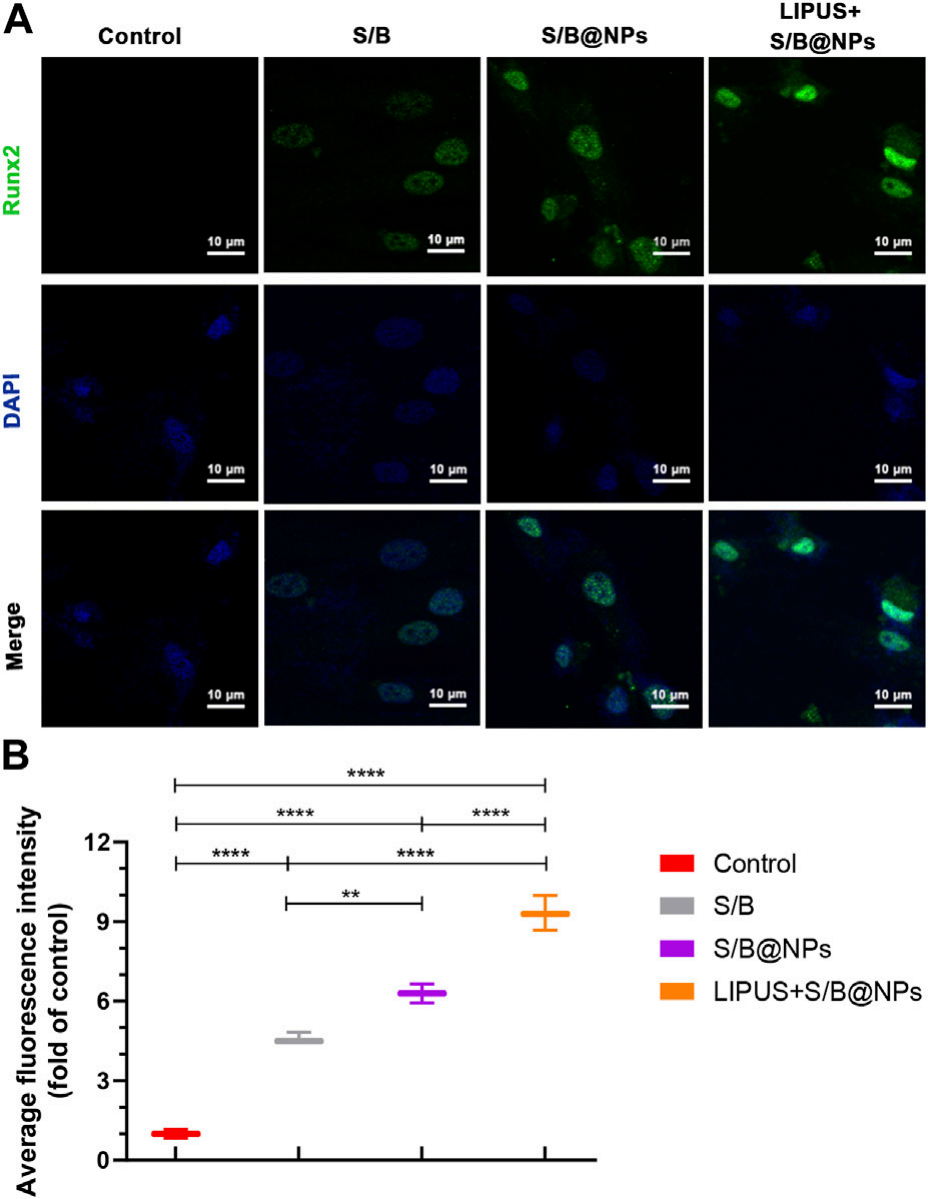
The effect of LIPUS-S/B@NPs on cell osteogenesis. **(A)** Immunofluorescence images stained with Runx2 (green) and DAPI (blue) and **(B)** its quantitative assessment. Error bar represents the mean ± SD (*n* = 3); *, *p* < 0.05; **, *p* < 0.01; ***, *p* < 0.001; ****, *p* < 0.0001.

To further evaluate the osteoinductive capacity of the LIPUS-S/B@NPs-delivery system, RT–qPCR and immunofluorescence staining assays were performed to detect the expressions of representative osteogenesis-related genes and proteins. The LIPUS treated group showed the highest expressions of *ALP*, *Runx2*, and *Col1a1* among groups, while their expressions in the S/B@NPs group were slightly higher than those in the S/B group (Figures 4C–E). Runx2 is a critical transcription factor during the cell osteogenic differentiation process (Komori et al., 1997). Therefore, immunofluorescence staining was conducted to further detect its expression in the cell. As shown in Figures 5A, B, the LIPUS treated group displayed the strongest green fluorescence located mainly in the nucleus among the groups. These results indicated that the LIPUS-S/B@NPs system could effectively promote cell osteogenic differentiation *in vitro*.

### 3.5 LIPUS-S/B@NPs promote rBMSCs recruitment to the rat periodontal bone defect

**FIGURE 6:**
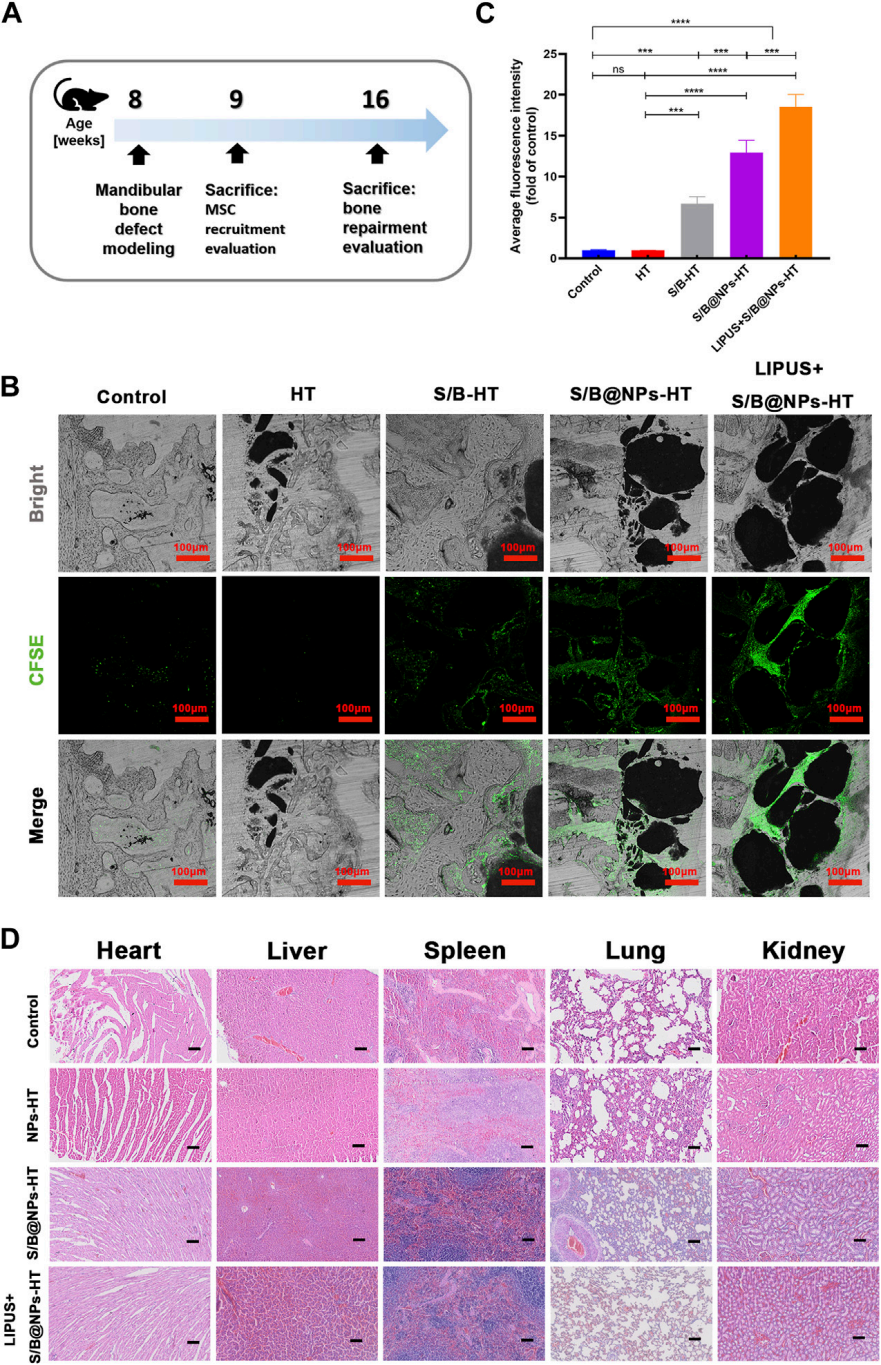
The effect of LIPUS-S/B@NPs on stem cell recruitment *in vivo* and its biocompatibility *in vivo*. **(A)** Schematic diagram illustrating the time frame of the *in vivo* study. **(B)** Confocal fluorescence images of hard tissue sections of periodontal bone defect area in rats to observe the recruited CFSE-labelled rBMSCs (green fluorescence) and **(C)** quantitative analysis of the fluorescence intensity. **(D)** H&E staining of heart, liver, spleen, lung, kidney tissues of SD rats after 8 weeks’ treatment. Scale bar: 100 μm. Error bar represents the mean ± SD (*n* = 3); *, *p* < 0.05; **, *p* < 0.01; ***, *p* < 0.001; ****, *p* < 0.0001.

With encouraging results *in vitro*, the stem cell recruiting and osteogenic capacities of the S/B@NPs delivery system were further evaluated in the rat periodontal bone defect model (Figure 6A). The rBMSCs were first labelled with CFSE probe *in vitro* for their further observation *in vivo*. The cells displayed bright green fluorescence, suggesting that they were successfully marked with CFSE. Then after the rat periodontal bone defect was prepared, the CFSE labelled rBMSCs were injected intravenously. After 1-week LIPUS treatment, the rats were sacrificed with their mandibles collected and sectioned to further evaluate the green fluorescence intensity of labelled rBMSCs migrated to the periodontal defect area. As shown in Figures 6B, C, brighter green fluorescence was observed in S/B, S/B@NPs and LIPUS + S/B@NPs groups than control and HT groups. Specifically, S/B@NPs group exhibited significantly stronger green fluorescence than S/B group; significantly, the LIPUS + S/B@NPs group displayed the brightest green fluorescence among all the groups.

### 3.6 *In vivo* toxicity evaluation of the LIPUS-S/B@NPs system

As shown in Figure 6D, the H&E staining results of viscera tissue displayed that there were no obvious pathological damages of main organs in all the experimental groups compared to that in the control group 8 weeks after the operation. It suggested that the systems displayed good bio-compatibility *in vivo*.

### 3.7 LIPUS-S/B@NPs promote bone regeneration in the rat periodontal bone defect

**FIGURE 7:**
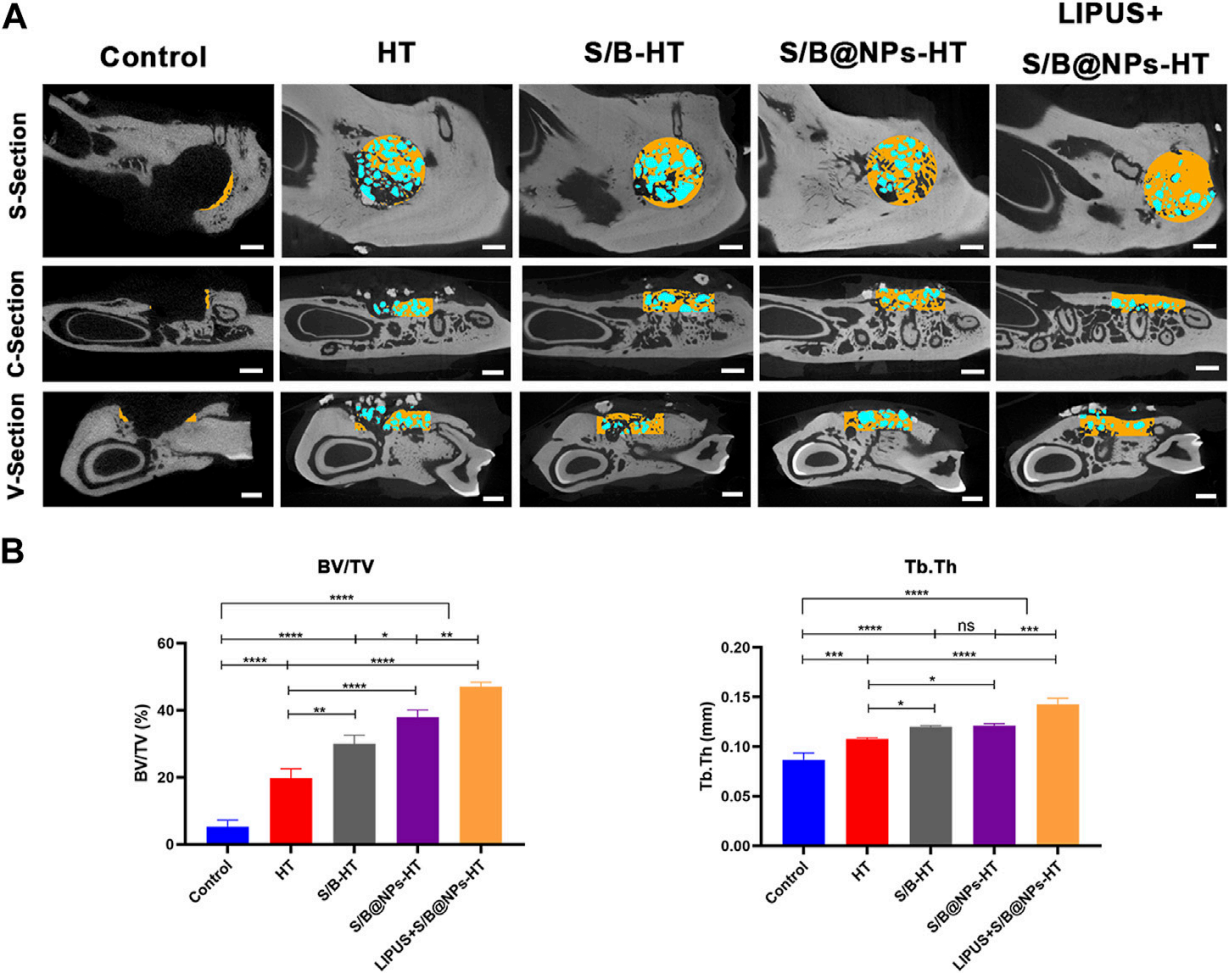
LIPUS-S/B@NPs promoted periodontal regeneration in rats. **(A)** Sagittal, vertical and cross-section micro-CT scanning images of rat periodontal bone defects. The new bone in the defect area is marked in yellow and the HT particles in blue. Scale bar: 1 mm. **(B)** Quantitative analysis of bone microarchitectural parameters determined by micro-CT. Error bar represents the mean ± SD (*n* = 3); *, *p* < 0.05; **, *p* < 0.01; ***, *p* < 0.001; ****, *p* < 0.0001.

Figure 7A shows the sagittal, vertical and cross sectional images of typical samples in each group; new bone in the defect area is marked in yellow and the implanted HA/TCP marked in blue. The control group displayed the least new bone formation and HT group displayed slightly more new bone. S/B@NPs-HT group exhibited slightly more bone regeneration than S/B-HT group. Among all the groups, LIPUS + S/B@NPs-HT group displayed the most bone regeneration. Moreover, there was less blue area in the LIPUS + S/B@NPs-HT group than other groups because the implanted HT particles, in our opinion, started to be replaced by newly-formed bone tissues. The results of bone volume fraction (BV/TV) evaluation were basically consistent with those of sectional images (Figure 7B). Microarchitectural parameters are important indexes that reflect the quality of new bone (Kimelman-Bleich et al., 2009). As shown in Figure 7B, the LIPUS + S/B@NPs-HT group displayed higher trabecular thickness (Tb. Th) than the other groups, indicating that the defect in LIPUS treated group did not only exhibited the most new bone formation, but also the highest bone quality.

**FIGURE 8:**
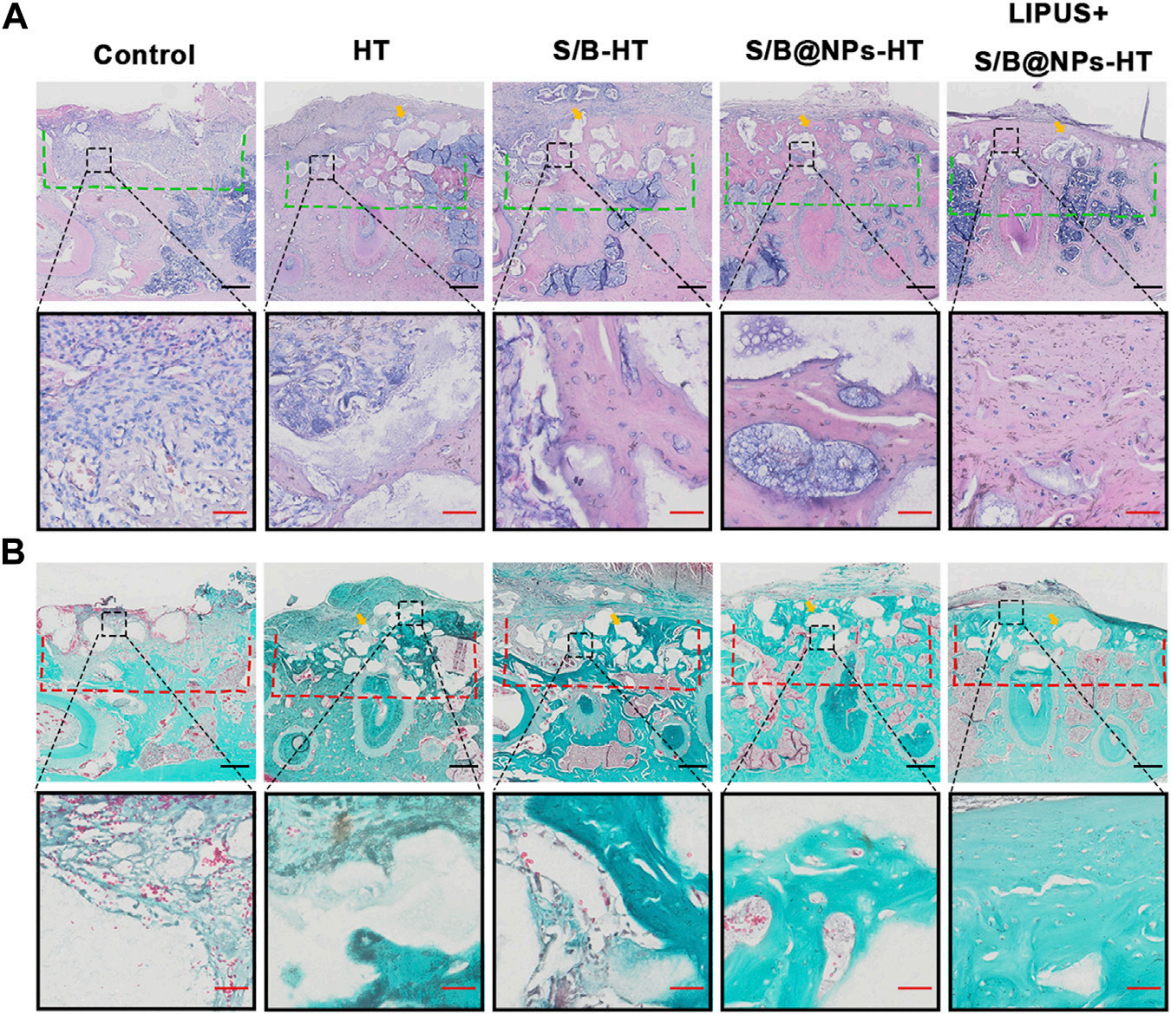
LIPUS-S/B@NPs promoted periodontal regeneration in rats. **(A)** H&E staining images of periodontal bone tissue sections. The defect area is marked with a green dotted line. **(B)** Masson-trichrome staining images of periodontal bone tissue sections. The defect area is marked with a red dotted line. Scale bar in black: 500 μm; scale bar in red: 50 μm. The yellow arrows refer to the implanted HT particles area.

H&E and Masson’s trichrome staining was employed to provide further complementary details regarding the defect area. As shown in Figures 8A, B, consistent with the results of micro-CT scanning, the defect area in the control group exhibited little new bone and was mostly filled with fibrous connective tissues. The HT group also displayed little new bone formation and defect was mostly filled with green staining collagen fibers. S/B-HT and S/B@NPs-HT groups displayed much more bone regeneration than the control group, in which the S/B@NPs-HT group had more new bone formation than S/B-HT group. Although the defect in S/B@NPs-HT group was mostly filled, most HT particles (indicated by yellow arrow) still existed without signs of bone remodeling initiation. The LIPUS + S/B@NPs-HT group showed much more new bone formation than the other groups, in which the defect was filled with newly formed woven bone with a well-arranged structure. Moreover, there were less HT particles (indicated by yellow arrow) in the defect area, which were completely surrounded by new bone. This indicated that the HT particles were starting to be replaced by new bone tissues, which usually happens in the late stage of bone repair. In a word, LIPUS + S/B@NPs-HT group displayed more mature bone tissues in the defect area, suggesting that our system could effectively promote periodontal bone regeneration.

## 4 Discussion

Nowadays, natural bone tissue regeneration is increasingly considered a multistage and well-choreographed process, in which each stage requires specific biological signals (Xu et al., 2019; Ying et al., 2020). Inspired by the natural bone healing cascade, a biomimetic LIPUS assisted drug delivery system was successfully designed and fabricated in this study, which could dually release SDF-1 and BMP-2 coupling the stem cell recruitment and osteogenesis processes. In this system, the combination of LIPUS can not only facilitate cell migration and osteogenesis, but also regulate the release of SDF-1 and BMP-2 in NPs. This system successfully promotes periodontal bone defect regeneration in SD rats.

*In situ* bone regeneration takes full advantage of the host proficiency by utilizing the cell-free biomaterials loaded with bioactive molecules to recruit cells from the surroundings or the circulation to the target area and initiate their osteogenesis *in situ*, which avoids the potential risks of traditional cell-loading tissue engineering strategy (Vaquette et al., 2018). In this study, the LIPUS-S/B@NPs system could effectively promote the rBMSCs migrating to the defect area in the early stage of bone repair, which could not only be attributed to its promotion effect on SDF-1 releasing from NPs, but also its physical effect on innate cell mechanism acting on cell migration. The early recruitment of stem cells could lay a solid foundation for further cell osteogenesis and bone repair process (Mehta et al., 2012; Martino et al., 2015). On the other hand, SDF-1 and BMP-2 have been widely used to recruit stem cells and promote osteogenesis in previous studies (Wegman et al., 2015; Shen et al., 2016; Wang T. et al., 2018b). It has been demonstrated that the combined utilization of SDF-1 and BMP-2 could achieve a synergistic effect over cell migration and osteogenesis, which is consistent with our results that the system releasing SDF-1 and BMP-2 significantly promoted stem cell recruitment and osteogenesis. Inspired by the natural bone healing cascade, Tan et al. (2019) prepared a supramolecular SDF-1/BMP-2/NapFFY hydrogel to promote rBMSC migration and osteogenesis in periodontal defect. 8 weeks after the implantation of the SDF-1/BMP-2/NapFFY hydrogel in the bone defect areas of rats, the bone regeneration rate of bone volume fraction reached 56.7%. Moreover, previous studies have demonstrated that the synergistic effect of combined utilization of SDF-1 and BMP-2 over cell migration and osteogenesis might be related to the activation of MAPK and Erk signaling pathways (Hwang et al., 2015). Therefore, in this study, a drug delivery system loaded with SDF-1 and BMP-2 was designed to couple the stem cell recruitment and osteogenesis cascade simulating the natural bone healing process. And the results of *in vivo* study displayed that such a biomimetic strategy could effectively promoted periodontal bone regeneration.

PLGA nanoparticles are widely used bio-material candidate for drug delivery systems for its good biocompatibility and biodegradation properties as well as the controllable drug-releasing rate (Ding and Zhu, 2018; Zhao et al., 2019). Growth factors like SDF-1 and BMP-2 are unstable and easily degraded, and require long-term and repeated administrations to maintain the effective concentration of growth factors *in vivo* (Lee et al., 2023; Zhang et al., 2023). Moreover, intravenous administration of growth factors easily leads to their accumulation in untargeted organs (like liver and kidney), causing unnecessary toxicity and side effects. Considering these, their encapsulation with PLGA nanoparticles can protect their bioactivity and sustain their release, resulting in prolonged efficacy (Revdekar and Shende, 2021; Heredia et al., 2022). As demonstrated in this study, our results showed that the S/B@NPs group displayed better cell migration and osteogenesis effects than the S/B group, indicating that the encapsulation of growth factors with NPs could promote their efficacy.

In this study, the combination of LIPUS in our system effectively promotes the bone repairing effect of S/B@NPs. Appropriate intensities of physical stimulation can enhance cellular metabolism and phenotypic adaptation (Flück, 2006). LIPUS is a form of mild and rhythmic mechanical energy with intensity ranging from 30 to 100 mW/cm^2^. Such a mechanical wave is known to transmit the living tissues and cells with “non-thermal effects,” which subsequently causes a lot of downstream pathway activation and biological effects at the cellular level (O'Brien, 2007; Rego et al., 2012). It has been approved for the treatment of fresh bone fractures by the Food and Drug Administration (FDA) in 1994 and widely applied in clinic as a safe and effective bio-physical therapy (Romano et al., 2009; Assanah et al., 2023). Moreover, several studies have demonstrated that LIPUS can upregulate the expressions of genes concerning mineral metabolism, cementoblastic differentiation and fibroblast differentiation (Inubushi et al., 2008; Mostafa et al., 2009). LIPUS has also proven to be able to effectively facilitate the hPDLCs osteogenesis and migration (Wang Y. et al., 2018c; Liu et al., 2020; Ying et al., 2020). Apart from the therapeutic effect onto cellular metabolism, ultrasound has been reported to regulate the release of drugs in specific carriers. In a previous study, the researchers used LIFU to trigger drug release in nanodroplets and enhance anticancer drug delivery (Yang et al., 2022). Another study demonstrated that VEGF loading microbubbles in combination of ultrasound targeted microbubble destruction could promote bone regeneration and vascularization at calvarial bone defects, which held huge potential for clinical translation (Gong et al., 2019). In this study, LIPUS with a power of 100 mW/cm^2^ was used. Compared with the ultrasonic wave used in previous studies, the application of LIPUS featuring low intensity and pulsed emission is mild, safe and can avoid the possible side effects of ultrasound upon bone fracture healing to a great extent, such as thermal effect. For the first time, our study found that LIPUS could effectively improve the release of S/B@NPs. It improved the unstable release rate of drugs loaded in NPs and effectively increased their final amount releasing from NPs, which, finally, resulted in greatly enhanced periodontal regeneration *in vivo*. In a word, our system can efficiently deliver BMP-2 and SDF-1 to the targeted area for bone regeneration, reducing the frequency of drug administration, their toxicity and side effects. Considering the dynamic/changing demands of bio-signals throughout the natural bone healing process, the LIPUS might be applied to regulate the release of drugs in osteogenesis-targeted drug delivery systems to fulfill the changing demands of bio-signals and maximize the bone regeneration efficiency in the future.

## 5 Conclusion

*In situ* tissue regeneration utilizes the cell-free biomaterials loaded with bioactive molecules to recruit the host stem cells and initiate their osteogenesis *in situ*, which can avoid the potential risks of traditional cell-loading tissue engineering strategy. In this study, we designed and manufactured a biomimetic LIPUS assisted drug delivery system that can dually release SDF-1 and BMP-2 to couple stem cell recruitment and osteogenesis of natural bone healing. In the system, LIPUS with a mild intensity of 100 mW/cm^2^ effectively avoids the side effects of ultrasound on fracture healing, and it can not only regulate the release of SDF-1 and BMP-2, but also promote cell migration and osteogenesis itself. The results showed that our system can significantly promote hPDLCs migration and osteogenesis *in vitro*; further *in vivo* evaluation displayed that it can effectively facilitate cell recruitment to target area and promote periodontal bone regeneration in SD rats. Natural bone healing is well-regulated and multistage process. For the first time, our study revealed that LIPUS can effectively regulate the release of SDF-1/BMP-2 from PLGA NPs. Considering its reliable safety and therapeutic effect on fracture, LIPUS, as an adjuvant therapy, holds great potential in the regulation of drug delivery systems for bone healing.

## Data Availability

The raw data supporting the conclusion of this article will be made available by the authors, without undue reservation.
